# Phosphatase and Tensin Homolog in Non-neoplastic Digestive Disease: More Than Just Tumor Suppressor

**DOI:** 10.3389/fphys.2021.684529

**Published:** 2021-06-01

**Authors:** Tianyu He, Xiaoyun Zhang, Jianyu Hao, Shigang Ding

**Affiliations:** ^1^Department of Gastroenterology, Peking University Third Hospital, Beijing, China; ^2^Department of Gastroenterology, Beijing Chao-Yang Hospital, Capital Medical University, Beijing, China

**Keywords:** PTEN, tumor suppressor, PI3K/AKT pathway, digestive organs, infection, inflammation, fibrosis

## Abstract

The Phosphatase and tensin homolog (*PTEN*) gene is one of the most important tumor suppressor genes, which acts through its unique protein phosphatase and lipid phosphatase activity. PTEN protein is widely distributed and exhibits complex biological functions and regulatory modes. It is involved in the regulation of cell morphology, proliferation, differentiation, adhesion, and migration through a variety of signaling pathways. The role of PTEN in malignant tumors of the digestive system is well documented. Recent studies have indicated that PTEN may be closely related to many other benign processes in digestive organs. Emerging evidence suggests that PTEN is a potential therapeutic target in the context of several non-neoplastic diseases of the digestive tract. The recent discovery of PTEN isoforms is expected to help unravel more biological effects of PTEN in non-neoplastic digestive diseases.

## Introduction

Phosphatase and tensin homolog (*PTEN*) gene, located on chromosome 10q23, was identified as a tumor suppressor gene approximately 20 years ago (Li et al., 1997). This gene modulates a wide range of biological processes by acting on both phosphoinositide and polypeptide substrates via multiple signaling pathways such as phosphatidylinositol 3-kinase (PI3K)/Akt/mammalian target of rapamycin (mTOR) (Maehama and Dixon, 1998; Manning and Cantley, 2007) and Ras/Raf/MEK/ERK (Hettinger et al., 2007). In addition, PTEN also acts through other non-enzymatic mechanisms. The phosphatase independent activity of PTEN contributes to the chromosomal stability and double-strand DNA breaks repair (Milella et al., 2015). Dysregulation of PTEN and its downstream signaling molecules may lead to abnormal cellular processes such as aberrant proliferation, abnormal survival, metabolism disorder, anomalous motility, and carcinogenesis.

Tumor, inflammation, infection, metabolic abnormalities, and fibrosis are common characteristics of organic diseases of the digestive system. *PTEN* mutation, *PTEN* loss, or its inactivity has been documented in the context of several cancers of the digestive tract (Eng, 2003; Bettstetter et al., 2013). However, since PTEN modulates a variety of biological processes, an increasing number of studies have investigated the changes in PTEN in the context of non-neoplastic digestive diseases such as hepatitis, colitides, pancreatitis, hepatic insulin resistance, and liver fibrosis. These studies have partially unraveled the potential role of PTEN and its dual phosphatase activity in non-neoplastic digestive diseases and the associated underlying mechanisms. In order to characterize the potential role of PTEN in the treatment of benign diseases, we reviewed the contemporary literature pertaining to PTEN protein published in the last decade, and highlight the potential role of PTEN in non-neoplastic digestive diseases.

## Structure and Activity of PTEN Protein

**FIGURE 1:**
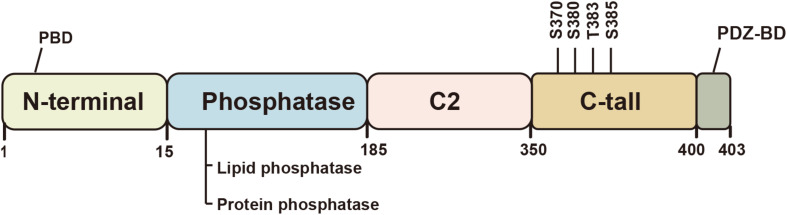
Schematic illustration of the structure of the PTEN protein. PTEN is a tumor suppressor with five functional domains: N-terminal phosphatidylinositol (Ptdlnsf4, 5) P2-binding domain (PBD), a phosphatase domain, a C2 lipid or membrane-binding domain, a carboxy-terminal tail, and a class I PDZ-binding (PDZ-BD) motif. The protein function largely depends on its lipid phosphatase and protein phosphatase activity. The C2 domain or C-terminal tail regulates the protein activity and stability.

PTEN protein consists of 403 amino acids and is encoded by the *PTEN* gene located on chromosome 10q23.31 (Li et al., 1997). Crystallographic analysis conducted in 1999 revealed its five functional domains, i.e., an N-terminal phosphatidylinositol (Ptdlns) (4,5) P_2_-binding domain (PBD), a phosphatase domain, a C2 lipid or membrane-binding domain, a carboxy-terminal tail, and a class I PDZ-binding (PDZ-BD) motif (Figure 1; Lee et al., 1999). The N-terminal domain is the main functional region of PTEN, which is homologous to tensin and auxilin, and is responsible for regulating the phosphatase activity by forming a wide substrate-binding pocket (Maehama and Dixon, 1998; Walker et al., 2004). The C-terminal domain is responsible for the activity, stability, and cellular localization of PTEN by regulating protein-protein interactions (Vazquez et al., 2000; Raftopoulou et al., 2004).

Phosphorylation of the C-terminal tail regulates the conformation of PTEN, which can affect the activity and stability of the PTEN protein. Unlike most proteins, the changes in PTEN function after phosphorylation may seem contradictory. Phosphorylation of S370 and cluster Ser380, Thr383, and Ser385 in the tail increases the stability of the protein and renders it less active (Fragoso and Barata, 2015). However, tail-deficient in PTEN causes loss of stability and confers phosphatase activity (Vazquez et al., 2000). This phenomenon has been shown to be associated with the transformation of closed and open conformation. The closed conformation is attributed to C-terminal phosphorylation, which promotes the interaction between acidic tail and C2 domain, and appears to increase stability but decrease lipid phosphatase activity via inhibiting membrane binding of PTEN (Vazquez et al., 2001). In the open conformation, dephosphorylation of PTEN reverses this change, and allows PTEN recruitment to the membrane and its binding to the PDZ domain-containing proteins (Chia et al., 2015).

Two recent studies have suggested PTEN dimerization as a new working model for the function of PTEN protein (Papa et al., 2014; Heinrich et al., 2015). The homo-dimeric PTEN complexes are critical for the lipid phosphatase function of PTEN and are more active than PTEN monomer in PIP3 dephosphorylation. However, the hetero-dimerization of cancer-associated PTEN mutants with wildtype PTEN has a dominant negative effect in cancer (Papa et al., 2014). The underlying mechanism for this phenomenon was revealed by another study which found PTEN homo-dimers *in vitro*. Dephosphorylation of the PTEN-tail, which confers it a more open conformation, was shown to allow dimerization and stabilize the homo-dimer (Heinrich et al., 2015). These findings provide insights into the cellular function and the molecular mechanism of PTEN activity regulation, which may provide a novel approach for cancer prevention and treatment.

## Biological Functions and Regulation of PTEN

With dual specific phosphatase activity, PTEN protein acts on both phosphoinositide and polypeptide substrates. Phosphatidylinositol (PtdIns)-3,4,5-trisphosphate (PIP_3_), a component of the lipid membrane, is considered to be the main substrate of PTEN (Maehama and Dixon, 1998). Dephosphorylation of this lipid substrate induces an inhibitory effect of PTEN on the PI3K-AKT-mTOR signaling pathway which is involved in cell growth, proliferation, survival, and metabolism (Manning and Cantley, 2007; Lee et al., 2018). In addition, PTEN can also cause dephosphorylation of some protein substrates, including focal adhesion kinase 1 (FAK), Shc, insulin receptor substrate-1 (IRS1), and cAMP-responsive element-binding protein 1 (CREB1) (Gu et al., 1999, 2011; Schneider et al., 2011; Shi et al., 2014). As both FAK and Shc are involved in integrin signaling, inhibition of their tyrosine phosphorylation by PTEN suppresses the integrin-mediated cell migration, which is independent of its effect on the PI3K/AKT pathway (Schneider et al., 2011; Zhang et al., 2014). Besides, PTEN selectively dephosphorylates IRS1, a mediator in insulin signaling, which plays a key role in metabolic diseases (Shi et al., 2014). Despite the discovery of an increasing number of polypeptide substrates of PTEN, further studies are required to characterize the biological roles of the dual phosphatase activity of the PTEN protein.

In addition to cytoplasm, PTEN is also found in nucleus, mitochondria, and endoplasmic reticulum (ER). The nuclear pool of PTEN is generally believed to exert its effects in a lipid phosphatase-independent way (Lindsay et al., 2006). Nuclear PTEN has been shown to play a role in DNA repair and maintenance of genomic stability (Shen et al., 2007). Additionally, it may also act as a pro-apoptotic factor by regulating cell cycle via suppressing cyclin D1 activity or by directly binding to the anaphase-promoting complex (APC/C) E3 ligase (Song et al., 2011). However, the underlying mechanisms are not well characterized. Presence of PTEN in mitochondria and ER was shown to be associated with cell apoptosis (Zhu et al., 2006; Bononi et al., 2013). ER-localized PTEN was found to enhance the transfer of calcium (Ca^2+^) from the ER to mitochondria and induce apoptosis. In addition, the interaction with inositol 1,4,5-trisphosphate receptors (IP3Rs) and the related Ca^2+^ release were involved in this process (Bononi et al., 2013). Moreover, PTEN-Long, a membrane permeable lipid phosphatase secreted from cells, was recently discovered as a variant of PTEN (Hopkins et al., 2013). It was also found to exhibit tumor suppressor effect after entering cells. A recent study demonstrated the localization of PTEN-Long at the outer mitochondrial membrane where it negatively regulated mitophagy by increasing the expression of PTEN-induced putative kinase protein 1 (PINK1) (Wang L. et al., 2018).

Several isoforms of PTEN have been identified in recent years. PTENα (also termed as PTEN-Long) is the first characterized isoform of canonical PTEN. It translates from a CUG start codon, and adds an alternatively translated region (ATR) at the N-terminus of PTEN (Hopkins et al., 2013). PTENα acts as a mitochondrial protein in mitochondrial bioenergetics (Wang L. et al., 2018). Another isoform of PTEN, PTENβ, whose translation is initiated from an AUU codon upstream of and in-frame with the AUG initiation sequence for canonical PTEN. It is mainly distributed in the nucleus and regulates pre-rRNA synthesis and cellular proliferation (Liang et al., 2017). PTENε (also termed as PTEN5) is a novel N-terminal-extended PTEN isoform initiated from the CUG816 codon within the 5′UTR region of PTEN mRNA, which suppresses tumor invasion and metastasis (Zhang Q. et al., 2021). Interestingly, a recent study related to PTEN isoforms demonstrated that PTENα and PTENβ can promote tumorigenesis via recruiting WDR5, but not regulation of AKT, to promote trimethylation of H3K4 and induce activation of relative oncogenes (Shen et al., 2019). This indicates that PTEN gene may act like a double-edged sword in the tumorigenesis process. Therefore, further studies are required for in-depth characterization of the role of the *PTEN* gene.

PTEN expression is regulated by various molecular mechanisms, including genetic alterations, epigenetic modifications, transcriptional post-transcriptional regulation, and post-translational regulation.

As a tumor suppressor gene, genetic loss or mutations of *PTEN* have been demonstrated in many primary human cancers and cancer cell lines (Guldberg et al., 1997; Ying et al., 2011). Early studies have demonstrated germline mutations of *PTEN* gene in *PTEN* hamartomatous tumor syndrome and Cowden syndrome; subsequently, more novel mutations have gradually been discovered in recent years (Liaw et al., 1997; Seol et al., 2015; Williams et al., 2018). Additionally, epigenetic modifications (such as DNA methylation and histone acetylation) may also regulate the expression of *PTEN*. *PTEN* loss caused by aberrant hypermethylation of the DNA promoter region has been identified in various malignant and benign (non-tumor) diseases (Mueller et al., 2012; Zhang et al., 2016; Geybels et al., 2017). Moreover, histone acetylation induced by the interaction of transcription factor SAL-like protein 4 and NuRD (a histone deacetylase repressor complex) at the promoter region can regulate *PTEN* transcription (Lu et al., 2009). In addition, inhibition of histone deacetylase (HDAC) has been shown to suppress the growth and invasion of cancer cells, which may provide a basis for novel anticancer therapies (Meng et al., 2016; Qian et al., 2018). Various transcription factors can upregulate or downregulate the expression of PTEN via multiple signaling pathways. For instance, p53, early growth response 1 (EGR1), and peroxisome proliferators activated receptor γ (PPARγ) can activate *PTEN* transcription via directly binding to the promoter region of *PTEN* (Stambolic et al., 2001; Lee et al., 2006; Kim et al., 2014); on the contrary, the ecotropic virus integration site 1 protein homologue (EVI1), B-lymphoma Mo-MLV insertion region 1 (BMI1) protein, and Ras/Raf/MEK/ERK pathway were shown to play a role in suppressing *PTEN* expression (Song et al., 2009; Yoshimi et al., 2011; Ciuffreda et al., 2012). Furthermore, at the post-transcriptional level, PTEN mRNA is regulated by microRNAs (miRNA; miRNA-21, -32, and -106b) (Wu et al., 2013, 2017c; Yang et al., 2014) and Long non-coding RNAs (LncRNA; LncRNA FER1L4, MEG3, and GAS5, etc.) (Qiao and Li, 2016; Gao et al., 2017; Wang J. et al., 2018). Moreover, some of the miRNAs and LncRNAs participate in the complex competing endogenous RNAs (ceRNAs) networks, represented by the mRNA of PTEN pseudogene 1 (PTENP1) and Versican (Lee et al., 2010; Poliseno et al., 2010). In addition, PTEN protein is regulated by phosphorylation, ubiquitination, sumoylation, acetylation, and redox regulation at the post-translational level (Worby and Dixon, 2014). Collectively, these findings illustrate the complex and diverse mechanisms of PTEN regulation, and lay the groundwork for therapeutic strategies against PTEN-associated diseases.

## PTEN and Non-Neoplastic Digestive Disease

**FIGURE 2:**
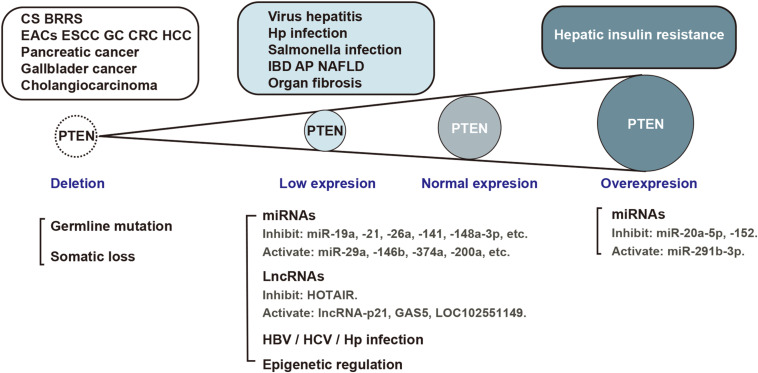
PTEN expression in the pathological process of digestive system diseases. Aberrant PTEN expression is implicated in several diseases of the digestive system. Gene mutation, translational or post-translational regulation is involved in the pathological process. EACs, esophageal adenocarcinomas; ESCC, esophageal squamous cell carcinoma; GC, gastric cancer; CRC, colorectal carcinoma; HCC, hepatocellular carcinoma; IBD, inflammatory bowel disease; AP, acute pancreatitis, NAFLD, non-alcoholic fatty liver disease; HBV, hepatitis B virus; HCV, hepatitis C virus; Hp, *helicobacter pylori*.

**FIGURE 3:**
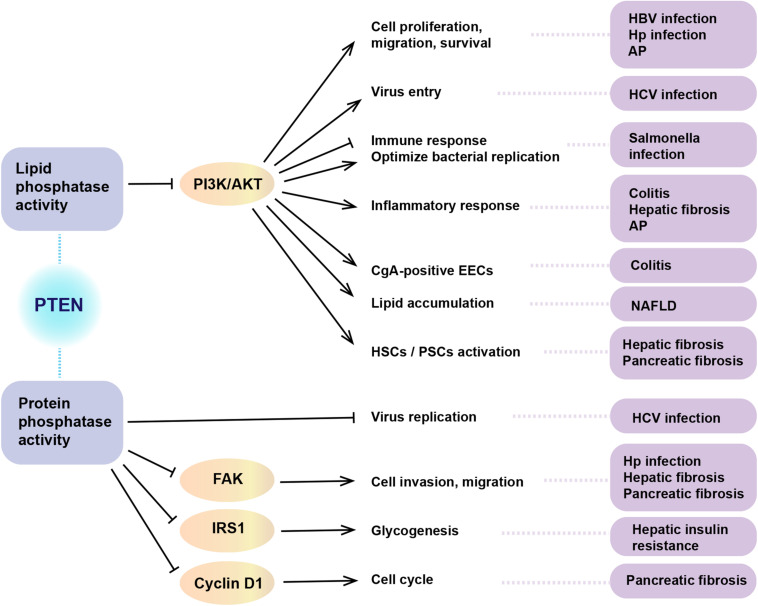
The role of dual phosphatase activity of PTEN in non-neoplastic digestive diseases. PTEN acts in regulating a wide spectrum of biological functions. The above summarizes the biological role of PTEN in the pathogenesis of non-neoplastic digestive diseases via lipid or protein phosphatase activity. HBV, hepatitis B virus; HCV, hepatitis C virus; Hp, helicobacter pylori; AP, acute pancreatitis; NAFLD, non-alcoholic fatty liver disease; HSC, hepatic stellate cells; PSCs, pancreatic stellate cells; CgA, chromogranin A; EECs, enteroendocrine cells; PI3K, phosphatidylinositol 3-kinase; FAK, focal adhesion kinase 1; IRS1, insulin receptor substrate-1.

The protein encoded by the *PTEN* gene is ubiquitous in the human body, including in the organs of the digestive system. As a critical tumor suppressor, mutation or deficiency of *PTEN* gene contributes to tumorigenesis in the digestive system, including esophageal carcinoma (Eng, 2003), gastric cancer (GC) (Wadhwa et al., 2013), colorectal carcinoma (CRC) (Ling et al., 2015), hepatocellular carcinoma (HCC) (Horie et al., 2004), pancreatic cancer (Ni et al., 2017), gall bladder cancer (Roa et al., 2015), and cholangiocarcinoma (Lee et al., 2012). However, studies conducted in recent years have demonstrated the regulatory role of PTEN in non-neoplastic diseases of the digestive system (Figure 2). It is increasingly being acknowledged that the biological role of PTEN in the pathogenesis of non-neoplastic digestive diseases may be mediated via mechanisms other than the dephosphorylation of PIP3 (Figure 3). In particular, many miRNAs and LncRNAs have been found to regulate the expression of PTEN in the pathogenesis of digestive system diseases (Table 1), laying an increasing emphasis on their role in gene regulation.

**TABLE 1:** The influence of microRNAs and long non-coding RNAs on PTEN in non-neoplastic digestive diseases.

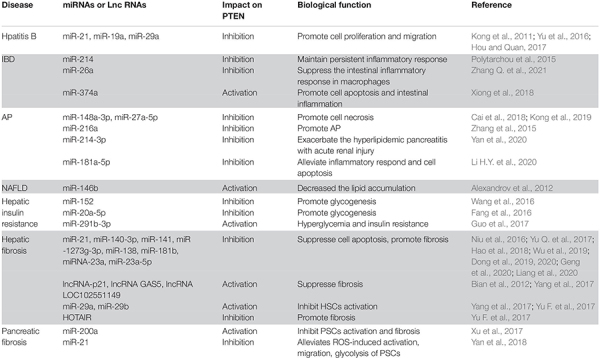

**miRNAs, microRNAs; lincRNA, long non-coding RNAs; IBD, inflammatory bowel disease; AP, acute pancreatitis; NAFLD, non-alcoholic fatty liver disease; HSCs, hepatic stellate cells; HOTAIR, homeobox transcript antisense RNA; PSCs, Pancreatic stellate cells; ROS, Reactive oxygen species.**

### PTEN in Infectious Diseases of Gut

#### Hepatitis B and C Virus Infection

Hepatitis B virus (HBV) and Hepatitis C virus (HCV) are major causes of viral hepatitis, and contributors to the incidence of HCC. Hepatitis virus induces dysregulation of hepatocyte metabolism; in addition, immune evasion of these viruses contributes to the development of chronic virus infection. Several studies have indicated the involvement of PTEN in these processes.

HBV X protein (HBx) is a key regulatory protein among the four proteins encoded by the HBV genome. Although HBx does not directly bind to the DNA, it influences the transcriptional activity of genes through interacting with the transcription factors or molecules of signaling pathways in host cells (Park et al., 2020). PI3K/AKT is one of the pathways activated by HBx, which regulates cell proliferation, cell death, and survival. Therefore, HBx is believed to play a pivotal role in the pathogenesis of HCC (Chung et al., 2004). PTEN is a specific inhibitor in the PI3K/AKT pathway; HBx downregulates PTEN, which promotes the proliferation and migration of liver cancer cells (Tu et al., 2019). Studies have identified several mechanisms by which HBx regulates the *PTEN* gene, including by influencing the epigenetic alterations of *PTEN* gene promoter, upregulating PTEN-targeted miRNAs (miRNA-21, miR-19a, miR-29a), and by promoting the production of reactive oxygen species (ROS) to inactivate PTEN (Ha and Yu, 2010; Kong et al., 2011; Um et al., 2011; Yu et al., 2016; Hou and Quan, 2017). Due to the regulation and alteration of the pro-apoptotic ability of PTEN by HBx, CRISPR/Cas9-mediated p53, and Pten somatic mutation was shown to accelerate hepatocarcinogenesis in adult HBV transgenic mice (Liu et al., 2017). In addition, PTEN has also been considered as a potential prognostic marker in patients with virus-induced HCC (Khalid et al., 2017). In addition, the interactions between PTEN polymorphisms and HBV mutations may help identify individuals who are susceptible to HCC (Du et al., 2015). HBx and HBV polymerase (HBp) were shown to enhance PD-L1 expression through PTEN-dependent pathway, which induces inhibition of T cell response and promotes virus immune evasion in mice (Sun et al., 2020).

PTEN is also a target pathogenic pathway of hepatitis C virus core protein. HCV core protein, an important agent related to HCC, can inhibit PTEN expression and promote virion egress (Clément et al., 2019). Studies have demonstrated that the dual phosphatase activity of PTEN has a protective effect against HCV infection by acting at different stages of its pathogenesis. The lipid phosphatase activity of PTEN inhibits HCV entry, while the protein phosphatase activity of PTEN helps decrease HCV replication through the interaction between domain I of HCV core and PTEN residues. In addition, HCV infection in turn increases the lipid phosphatase activity of PTEN (Wu et al., 2017b). In human hepatocyte-engrafted (MUP-uPA/SCID/Bg) mice model, PTEN depletion was shown to play an important role in the initiation of HCV infection-associated HCC (Wang et al., 2015b). Based on the role of PTEN in the process of HCV infection, PTEN-Long, a translation isoform of PTEN, may inhibit HCV replication by interacting with the HCV core protein (Wu et al., 2017a). Therefore, exogenous administration of PTEN is a potential therapeutic strategy against viral hepatitis.

#### *Helicobacter pylori* Infection

*Helicobacter pylori* (*H. pylori*) infection is the main risk factor for GC. Chronic *H. pylori* infection contributes to chronic non-atrophic gastritis, which eventually develops into dysplasia and ultimately GC. Only a few studies have focused on the involvement of PTEN in *H. pylori* infection. GC patients with *H. pylori* infection showed significantly decreased expression of *PTEN* gene in serum as compared to healthy volunteers; this suggested a potential role of PTEN gene in GC patients with *H. pylori* infection (Ranjbar et al., 2018). A series of studies investigating the role of PTEN in the pathogenesis of *H. pylori* infection yielded interesting findings. First, phosphorylation of PTEN at residues Ser380/Thr382/383 induced by *H. pylori* was found to promote the survival of gastric epithelial cells, since the phosphorylation of PTEN leads to the loss of phosphatase activity and activation of the PI3K/AKT pathway (Yang et al., 2015). Subsequently, PLK1 were found to influence p-PTEN level, which may be involved in the early stage of *H. pylori*-induced GC (Xu et al., 2018). Another study also found that *H. pylori* can promote cell invasion via phosphorylation of PTEN and activation of FAK (Yang et al., 2018). Further studies are required to clarify the mechanisms of PTEN regulation in *H. pylori* infection and *H. pylori*-induced GC.

#### Salmonella Infection

*Salmonella enterica* (Gram-negative enteropathogenic bacteria) is one of the most common causes of food poisoning. People are generally susceptible to Salmonella, especially infants and elderly people; the clinical manifestations depend on the virulence of the strain and the immune status of the host (Kurtz et al., 2017). PTEN has been considered vital for Toll-like receptor (TLR) 5-induced immune and inflammatory responses in the intestinal epithelial cells (IECs). PTEN deficiency in IECs can increase the susceptibility to Salmonella infection (Howe et al., 2019). Besides, when challenged with Salmonella infection, PTEN was epigenetically suppressed by CUL4B, which negatively regulates the TLR-triggered signaling and maintains the anti-inflammatory pathway PI3K-AKT-glycogen synthase kinase (GSK) 3β. However, overexpression of PTEN caused by CUL4B deletion contributed to excessive activation of GSK3 and uncontrolled immune response, which may increase the risk of septic shock in infected individuals (Song et al., 2021).

SopB is an important modulator of signal transduction with phosphatase activity in host cells during Salmonella infection. SopB activates pro-survival kinase Akt, which optimizes bacterial replication in host, while PTEN can inhibit Akt activation during Salmonella invasion (Roppenser et al., 2013). These findings indicate a vital role of PTEN in the process of Salmonella infection, including in determining the susceptibility to bacterial invasion and modulating the inflammatory response in host cells. However, further studies are required to unravel the precise mechanism of the involvement of PTEN in these processes.

### PTEN in Inflammation Disease of Gut

#### Colitides

Colitides is a generic term used to describe inflammatory diseases of colon, including inflammatory bowel disease (IBD)–associated colitis and non-IBD forms of colitis (such as microscopic colitis, radiation colitis, eosinophilic colitis, and ischemic colitis). PTEN is believed to play an important role in the inflammation associated with collagenous colitis (Norén et al., 2018) and IBD (Li et al., 2013), as PTEN deficiency has been documented during this process. Upregulation of interleukin (IL)-6 was shown to induce STAT3-mediated miR-214 expression in active ulcerative colitis (UC). miR-214 was shown to activate and amplify the inflammatory response through a feedback loop mediated by PTEN and PDZ and LIM domain 2 (PDLIM2) suppression, and with increased AKT phosphorylation and NF-κB activation (Polytarchou et al., 2015). A recent study conducted in miR-26a myeloid-cell-specific overexpression mice showed that miR-26a attenuates the intestinal inflammatory response in macrophages by inhibiting the activation of NF-κB/STAT3 and decreasing the production of IL-6. In addition, PTEN was identified as one of the potential targets of miR-26a (Zhang W. et al., 2021). The above findings indicate that PTEN reduction may contribute to the circuit activation and persistent inflammatory response in UC and even progression to CRC.

Mutation of gene encoding IL-10 is believed to play a role in the pathogenesis of early onset UC which is characterized by severe colitis in infancy and early childhood. IL-10(−/−) mice were shown to develop spontaneous colitis in the presence of intestinal microbiota; in addition, disruption or inhibition of PTEN was found to increase the severity of colitis and influence colitogenic bacteria in these mice. Due to the impact of PTEN on important cell functions and TLR signaling, disruption of PTEN in the intestinal epithelium of IL-10(−/−) mice hastened the occurrence of severe colitis. Moreover, increased abundance of Bacteroides species was detected in fecal microbiota of PTEN loss IL-10(−/−) mice (Im et al., 2014). On the other hand, inhibition of PTEN function in IL-10(−/−) mice was also shown to enhance the production of inflammatory factors and to increase the proportion of colitogenic bacteria (Bacteroides and Akkermansia) in fecal microbiome (Mitchell et al., 2018). However, on the contrary, PTEN deletion was found to attenuate colonic inflammation in IL-10(−/−) mice. Microbial factors play an essential role in the development of colitis. In a study, flagellin elicited colonic inflammation in IL-10(−/−) mice. However, deletion of PTEN was shown to disrupt Mal-TLR5 interaction and diminish the flagellin-promoted inflammatory responses by impeding Mal localization at the plasma membrane and preventing Mal-TLR5 interaction (Choi et al., 2013). The available evidence suggests that PTEN may mediate the microbe-induced inflammatory immune response in the intestines; however, the specific regulatory mechanisms are not clear.

IBDs are characterized by increased intestinal permeability, which is mainly attributable to the impaired integrity of the tight junctions. Decreased expressions of tight junction-related genes (such as *MAGI3, PTEN*, and *TJP1*) were observed in the colonic mucosa of patients with IBD (Norén et al., 2017). These findings support the role of *PTEN* gene in modulating the intestinal epithelial barrier function in IBD. In addition, increase in the enteroendocrine cells (EECs) in mucosa is another manifestation in IBD patients. Pro-inflammatory cytokines promote the number of EECs producing chromogranin A (CgA) along with inactivation of PTEN and increased expression of AKT and autophagy markers in EECs. However, inhibition of AKT and autophagy was found to block the increase in CgA-positive cells (Hernández-Trejo et al., 2016). These findings suggest that PTEN and downstream AKT signaling pathway as well as autophagy regulate the differentiation of EECs during colonic inflammation.

Currently, several drugs that regulate the activity and expression of PTEN are used in the treatment of IBD. 5-aminosalicylic acid (5-ASA), an important drug for treatment of UC, is a potent antioxidant. It can reduce the phosphorylated and oxidized levels of PTEN protein via inducing PPARγ binding to the PTEN promoter; this promotes the activation of PTEN and inhibition of the PI3K/Akt signaling pathway (Managlia et al., 2013). This ultimately reduces the UC-induced ROS in the IECs. In addition, fortunellin, which is isolated from the kumquat fruit, was shown to ameliorate IBD (Vezza et al., 2016). However, contrary to the effects of 5-ASA, fortunellin suppresses the expression of PTEN to reduce epithelial cell apoptosis and ameliorate the symptoms of colitis by targeting miR-374a in rats (Xiong et al., 2018). Chlorogenic acid, an abundant polyphenol in medicinal plants, has been demon- strated to have anti-inflammatory property. Upregulation of PTEN and suppression of Akt and STAT3 expression has been detected in dextran sulfate sodium-induced UC mice model (Vukelić et al., 2018). Thus, regulation of PTEN is a potential therapeutic target for treatment of intestinal inflammation.

#### Acute Pancreatitis

Acute pancreatitis (AP) is a necro-inflammatory disease. Severe acute pancreatitis (SAP) is associated with multiple organ insufficiency and high mortality. A series of studies have focused on the role of miRNAs in AP progression and treatment via regulating PTEN. miR-148a-3p and miR-27a-5p, which are highly expressed in AP, were shown to induce acinar cell apoptosis through targeting PTEN (Cai et al., 2018; Kong et al., 2019). In a mouse model of AP and in rat pancreatic acinar cells, transforming growth factor (TGF)-β was shown to upregulate miR-216a, which inhibited the expressions of PTEN and Smad7 and promoted AP via the PI3K/Akt and TGF-β feedback pathway (Zhang et al., 2015). SAP usually leads to multiple organ dysfunction, especially renal damage. Overexpression of miR-214-3p in a rat model of hyperlipidemic pancreatitis was shown to inhibit PTEN expression and up-regulate the level of P-Akt in kidneys to exacerbate hyperlipidemic pancreatitis with acute renal injury (Yan et al., 2020).

Bone marrow mesenchymal stem cells (BM-MSCs) have an anti-inflammatory effect and showed a protective effect in SAP (Zhao et al., 2016). miR-181a-5p secreted by BM-MSCs reduced the level of angiopoietin, IL-1β, IL-6, and TNF-α, and promoted the expressions of IL-4 and IL-10 to alleviate inflammatory response and cell apoptosis by targeting the PTEN/Akt/TGF-β1pathway (Li H. Y. et al., 2020). Rosiglitazone is widely used for the treatment of diabetes as it increases insulin sensitivity. In recent years, several studies have demonstrated the effects of rosiglitazone on inflammatory response and cell metabolism (Ji et al., 2018). Rosiglitazone was shown to prevent AP by downregulating miR-26a, inhibiting PTEN degradation, and blocking the PI3K/AKT signaling pathway in AP rats (Chen et al., 2019). Thus, an increasing body of evidence has demonstrated the important role of the interaction between miRNAs and PTEN in the context of AP, identifying novel potential therapeutic targets for SAP.

### PTEN in Metabolic Abnormalities of Digestive Organs

#### Non-alcoholic Fatty Liver Disease

Non-alcoholic fatty liver disease (NAFLD) is one of the most common chronic liver diseases. It is associated with increased risk of type 2 diabetes and cardiovascular diseases (Musso et al., 2011). The term NAFLD encompasses a spectrum of fatty liver diseases including simple steatosis, non-alcoholic steatohepatitis (NASH), and NASH-related fibrosis (Kleiner and Makhlouf, 2016). Different from alcoholic liver disease, patients with NAFLD exhibit decreased expression of PTEN in liver tissues; in addition, the degree of downregulation is associated with the percentage of steatosis (Sanchez-Pareja et al., 2016). Furthermore, in mice models, long-term exposure to high-fat diet decreased the PTEN expression in liver and led to mild-to-moderate NAFLD (Nalloor et al., 2017). PTEN deficiency is believed to accelerate the development of NAFLD (Jeong et al., 2018). Knock-down of PTEN in rat liver via CRISPR/Cas-based hydrodynamic injection of pX330-Pten plasmid has been used to construct rat model of NAFLD (Yu Q. et al., 2017).

Hypoxia is one of the factors that aggravate the NAFLD phenotype with increased lipogenesis and inflammation in PTEN-deficient mouse (Byrne, 2009). Another study reported the involvement of spleen in the pathogenesis of NAFLD. Splenectomy was shown to accelerate hepatic steatosis and to increase serum lipid levels through down regulating hepatic PTEN expression and promoting the ratio of pAkt/Akt (Wang et al., 2015a). On the contrary, PTEN upregulation was shown to alleviate NASH. Since miR-146b directly suppresses the IL-1 receptor-associated kinase 1 and tumor necrosis factor receptor-associated factor 6, the downstream molecules NF-κB and PTEN were down and up regulated, and decreased lipid accumulation in the liver cells (Jiang et al., 2015). The above results indicate that PTEN is a potential target for treatment of NAFLD.

#### Hepatic Insulin Resistance

Glucose homeostasis is achieved by balancing pancreatic insulin secretion with intake or secretion of glucose. Hepatic insulin resistance is defined as the impaired ability of hepatocytes to respond to insulin, which contributes to the progression of type 2 diabetes mellitus (T2DM) and metabolic syndrome (Leclercq et al., 2007). Binding of insulin with the insulin receptor induces tyrosine phosphorylation of insulin receptor substrates (IRS1/2). This process enhances the PIP2 to yield PIP3 via PI3K, which can be inhibited by PTEN. PIP3 contributes to membrane localization of PDK1 and Akt1, and induces Akt1 phosphorylation resulting in glucose uptake and utilization. A recent study showed that phosphorylating IRS-1 at Ser 307 can lead to inhibition of phosphorylation of Akt and GSK-3β and reverse the insulin resistance in HepG2 cells (Malik et al., 2019). Further study revealed the mechanisms by which endosomes activate the hepatic insulin-evoked Akt signaling pathway. The results demonstrated that the binding of IRS 1 and 2 (IRS1/2) and the endosomal insulin receptor (INSR) can promote IRS1/2 phosphorylation and initiate downstream Akt2/GSK-3β and FoxO1 signaling in the liver (Zhang et al., 2020).

Owing to the role of PI3K/Akt signaling in insulin function and glucose metabolism, increased expression of PTEN is believed to promote insulin resistance in liver (Alexandrov et al., 2012; Zuo et al., 2019). A series of studies have revealed aberrant expression of microRNAs associated with insulin resistance through regulating PTEN directly or indirectly. miR-152, which directly targets PTEN expression, was shown to be downregulated in the liver of high fat diet-fed mice, which resulted in increased expression of PTEN and subsequent impaired glycogenesis as well as hepatic insulin resistance. The AKT/GSK pathway was found to be involved in this process (Wang et al., 2016). In addition, miR-20a-5p suppresses p63 and in turn binds to p53, diminishing PTEN expression and participates in hepatic glycogen synthesis by activating AKT and GSK (Fang et al., 2016). However, miR-291b-3p augments PTEN expression and impairs AKT activation by targeting p65, leading to hyperglycemia and hepatic insulin resistance (Guo et al., 2017). A recent study investigated the molecular mechanism of PTEN regulation in balancing insulin action via an oxide transport chain NSAPP. NADPH oxidase-4 (NOX4), a part of insulin signaling NSAPP, generates superoxide (O_2_–) after being stimulated by insulin; NOX4 combines with superoxide dismutase-3 to transfer O_2_– converting it to hydrogen peroxide. Finally, aquaporin-3 transports H_2_O_2_ across the plasma membrane to inactivate PTEN. Thus, disruption of any molecule in the NSAPP chain may lead to persistent PTEN activation and imbalanced insulin action (Wu et al., 2020). In addition to liver, muscle, and adipose tissue, brain has been identified as a new target for insulin and a site for glucose metabolism. Insulin acts on the mediobasal hypothalamus (MBH) to improve the glycometabolism through PI3K activation. In addition, inhibition of PTEN activity in MBH was shown to decrease food intake and weight gain, and also regulate liver insulin resistant independently in high-fat-fed rats (Sumita et al., 2014).

HCV infection was shown to be a risk factor for insulin resistance and T2DM. Owing to the close relationship between viral hepatitis and NAFLD, steatosis is one of the characteristics of HCV infection. HCV genotype 3a core protein was shown to block PTEN translation via miRNA-dependent mechanism. The diminished expression of PTEN in turn led to reduced expression of IRS1 and formation of large lipid droplets (Clément et al., 2011). Similarly, upregulation of PTEN expression was shown to promote insulin sensitivity and reduce the release of proinflammatory factors in mice liver infected with HCV core protein (Jia et al., 2018). However, other studies indicated that liver steatosis is not always associated with insulin sensitivity (Matsumoto et al., 2006; Wu et al., 2012). Oleic acid (OA) was shown to induce hepatic steatosis but with normal insulin sensitivity; this was attributable to activation of the G protein-coupled receptor 40 (GPR40)-phospholipase C (PLC)-calcium pathway by OA and upregulation of PPARδ. PPARδ further reduced the expression of PTEN to enhance insulin sensitivity in hepatic steatosis.

### PTEN in Digestive Organ Fibrosis

#### Hepatic Fibrosis

Hepatic fibrosis, a prominent pathological feature of chronic liver disease, leads to liver cirrhosis, liver failure, and hepatic carcinogenesis. Activation of Kupffer cells (KCs) and hepatic stellate cells (HSCs) plays a pivotal role in this pathogenesis. A growing body of evidence supports the link between PTEN and hepatic fibrosis.

KCs refer to a type of tissue macrophages resident in liver. In a CCl4-induced mouse model of liver fibrosis, KCs showed mixed induction of hepatic classical (M1) and alternative (M2) macrophage markers. PTEN regulates the activation and polarization of M2 macrophages by inhibiting the PI3K/Akt/STAT6 signaling pathway to counteract liver injury (Cheng et al., 2017).

Deposition of extracellular matrix (ECM) caused by the activation of HSCs is a key process in the development of hepatic fibrosis. Hence, identification of potential targets to inhibit the activation of HSCs is a potential strategy for prevention and treatment of hepatic fibrosis. Several recent studies have focused on the role of regulatory non-coding RNAs, such as miRNAs and long intergenic non-coding RNAs (lincRNAs) in the activation of HSCs by affecting PTEN. MiR-21 (Hao et al., 2018), -140-3p (Wu et al., 2019), -141 (Liang et al., 2020), and -1273g-3p (Niu et al., 2016) have been shown to be negative regulators of PTEN, which lead to HSCs activation, cell apoptosis inhibition, and fibrosis formation through regulating the PI3K/AKT or AKT/mTOR pathway. Additionally, Dicer, an enzyme with endonuclease activity, is involved in cutting precursor miRNAs to produce functional forms. Among several miRNAs affected by dicer, miR-138, which targets PTEN, can be downregulated most significantly, resulting in altered collagen synthesis in HSCs (Yu et al., 2014). LincRNAs have also been shown to play a pivotal role in the regulation of biological behavior of HSCs and liver fibrosis. Since PTEN is the target of miR-181b, lincRNA-p21 promotes PTEN expression, inhibits HSCs activation and ECM deposition through competitive binding to miR-181b (Yu et al., 2014; Geng et al., 2020). Other studies have shown that lncRNA GAS5 (Dong et al., 2019) and lncRNA LOC102551149 (Dong et al., 2020) can decrease the expressions of miRNA-23a and miR-23a-5p, respectively, increase the level of PTEN, and suppress liver fibrosis via acting on the PI3K/Akt/mTOR/Snail signaling pathway.

PTEN expression is also regulated at the epigenetic level. DNA methyltransferase (DNMT) 1 mediated hypermethylation of PTEN promoter and loss of PTEN expression were shown to induce the activation of HSCs by influencing the PI3K/AKT and ERK pathways (Bian et al., 2012). In addition, miR-29a has been shown to decrease the expressions of DNMT1 and DNMT3b, reduce the methylation of PTEN, and inhibit the activation of HSCs (Yang et al., 2017). Hence, as promoters of miR-29a expression, curcumin (Zheng et al., 2014) and adiponectin (Kumar et al., 2018) can suppress the activation of HSCs and inhibit liver fibrosis by decreasing the methylation of PTEN CpG. In another study, homeobox transcript antisense RNA (HOTAIR), a lincRNA associated with attenuation of miR-29b’s epigenetic regulation and downregulation of PTEN expression through sponging miR-29b, promoted the development of liver fibrosis in CCl_4_ treated mice (Yu Q. et al., 2017).

The above findings strongly suggest a role of PTEN in inhibiting hepatic fibrosis. Administration of adenovirus encoding PTEN was shown to decrease collagen deposition in a rat model of liver fibrosis (Xie et al., 2017). This indicated that gene therapy using adenovirus-mediated PTEN is a potential novel therapeutic strategy for liver fibrosis.

#### Pancreatic Fibrosis

Pancreatic fibrosis is a key pathological finding in several pancreatic diseases including chronic pancreatitis, autoimmune pancreatitis, and cystic fibrosis of the pancreas. Activation of pancreatic stellate cells (PSCs) is a key step in the initiation of pancreatic fibrosis. PTEN has recently been recognized as a potential regulatory target for ameliorating pancreatic fibrosis. PTEN protein exhibits dual phosphatase activity. The wildtype PTEN was shown to be more effective than mutant (G129E) PTEN (which only has protein phosphatase activity) in inhibiting the proliferation and migration of PSCs and collagen synthesis (Zhang et al., 2018). PTEN was shown to regulate cell cycle by affecting p27Kip1 and cyclinD1 in activated PSCs; this indicates a potential role of nuclear PTEN in the activation of PSCs. The activity of cyclin D1 is required for cell cycle G1/S transition. PTEN can negatively regulate the cell cycle by suppressing cyclin D1in PSCs, which is consistent with lung fibroblasts (Geng et al., 2016) and hepatic stellate cells (An et al., 2016). The similar suppression ability of wildtype and mutant (G129E) suggests that PTEN mainly suppresses cyclin D1 via its protein phosphatase activity in PSCs. However, p27Kip1, which specifically binds to CDK-cyclin complexes to initiate cell cycle arrest, was upregulated by PTEN (Zhang et al., 2018). The role of PTEN in regulating cell cycle seems to be similar in different pro-fibrotic cell types. Besides, the protein expressions of BAX and Bcl-2 were up- and down-regulated, respectively, by PTEN-induced apoptosis of PSCs; thus, further studies are required to identify the step where mito-PTEN or the cellular localization changes of PTEN are involved in this process. In another study, miR-200a promoted the expression of PTEN and attenuated the TGF-β1-induced activation of PSCs and deposition of ECM in rat (Xu et al., 2017).

Some recent studies have shown that autophagy leads to the activation of PSCs (Xue et al., 2017; Li Z. et al., 2020). As a negative regulator of the PI3K-AKT-mTOR signaling pathway, PTEN is believed to play an important role in autophagy. However, the role of PTEN in regulating mytophagy during activation of PSCs and the underlying mechanisms are largely unknown. A study revealed that PTEN inhibition can promote the expression of Mitofusin-2 (Mfn2) and improve mitophagic flux via AMP-activated protein kinase (AMPK)-cAMP-response element-binding protein (CREB) signaling (Li P. et al., 2020). Phosphorylation of Mfn2 has been shown to dissociate mitochondria from ER, inducing initiation of mitophagy. Intriguingly, PTEN and Mfn2 are both localized at the ER-mitochondrial contact site (de Brito and Scorrano, 2008; Bononi et al., 2013). Thus, further studies should assess whether the interaction between PTEN and Mfn2 at ER-mitochondrial contact plays a vital role in the activation of PSCs.

Activated PSCs are also key precursor cells for cancer-associated fibroblasts and produce a microenvironment that enhances malignancy in pancreatic cancer. Silencing of miR-21, which targets PTEN, was shown to alleviate ROS-induced activation, migration, and glycolysis of PSCs and to inhibit pancreatic cancer cells simultaneously (Yan et al., 2018). In pancreatic ductal adenocarcinoma (PDAC) stroma, blocking the Hedgehog signaling in stromal fibroblasts induced proliferation of tumor cells, which was caused by PTEN degradation and AKT activation. Therefore, decreased stromal PTEN is associated with reduced survival in PDAC patients (Pitarresi et al., 2018). The above results indicate the crucial role of PTEN in pancreatic fibrosis. Further studies are required to unravel the underlying mechanisms.

## Conclusion and Perspectives

It has been recognized that subtle variations in *PTEN* gene, *PTEN* expression, or PTEN protein level have enormous consequences in terms of susceptibility to digestive system cancers. Advances in biotechnology have helped enhance our understanding of the biological functions of PTEN. An increasing body of evidence indicates that PTEN loss or inactivity is one of the major causes of cellular dysfunction in a broad spectrum of non-neoplastic digestive diseases, such as infection, inflammation, metabolic abnormalities, and fibrosis. Though these diseases seem to be benign, their long-term existence or repeated occurrence may trigger carcinogenesis. Hence, early alterations in PTEN and the degree of these changes in non-neoplastic diseases may warn against occurrence of cancer.

Along with the discovery of canonical and non-canonical PTEN, multiple sites of cellular localization and various molecular interactions of this protein have been identified. However, the difference of PTEN cellular localization and the shuttling process of PTEN between cytosol and nucleus during the development of non-neoplastic digestive diseases is not well characterized. Especially, PTEN-Long can be secreted from cells and taken up by other cells; its localization in mitochondria has been found to suppress mitophagy in central nervous system diseases and kidney inflammation. However, the role and mechanism of PTEN-Long and the effect of the changes in its cellular localization on the development of non-neoplastic digestive diseases is not well understood. Hence, the molecular mechanisms for PTEN in the mitochondria and the nucleus in the context of non-neoplastic digestive diseases should be further investigated.

Clinical trials of PTEN-targeted therapies have been conducted in patients with cancer (Bang et al., 2019) and Alzheimer’s disease (Mohamed et al., 2019). Recent years have witnessed rapid advances in gene therapy and its application in clinical settings (Dunbar et al., 2018). In particular, the CRISPR/Cas9 gene-editing technology has provided researchers with revolutionary tools for gene therapy. However, gene therapies targeting PTEN are still in the animal experiment stage. Due to the difference between the *in vivo* and *in vitro* environment, and the discrepancy between species, the results obtained from the cell or animal models have some inherent limitations. However, no clinical trials involving PTEN targeting have been conducted in patients with non-neoplastic digestive diseases. As PTEN is a potential therapeutic target in the context of several benign diseases of the digestive system, more work is required to unravel the related molecular mechanisms. Translation of research results into clinical application needs more time.

## Author Contributions

JH designed the manuscript. TH and XZ drafted the manuscript and drawn the figures and table. SD and JH review and edited the article. SD supervised the article. All authors read and approved the final manuscript.

## Conflict of Interest

The authors declare that the research was conducted in the absence of any commercial or financial relationships that could be construed as a potential conflict of interest.

## Abbreviations

PTEN: Phosphatase and tensin homolog
PIP3: phosphatidylinositol
PI3K: phosphatidylinositol 3-kinase
mTOR: mammalian target of rapamycin
PBD: phosphatidylinositol (4,5) P2-binding domain
PIP3: Phosphatidylinositol-3,4,5- trisphosphate
FAK: focal adhesion kinase 1
IRS1: insulin receptor substrate-1
CREB1: cAMP-responsive element-binding protein 1
ER: endoplasmic reticulum
IP3Rs: inositol 1,4,5-trisphosphate receptors
PINK1: PTEN-induced putative kinase protein 1
SALL4: SAL-like protein 4
HDAC: histone deacetylase
EGR1: early growth response 1
PPAR γ: peroxisome proliferators activated receptor γ
miRNA: microRNA
LncRNA: Long non-coding RNAs
ceRNA: complex competing endogenous RNA
PTENP1: PTEN pseudogene 1
GC: gastric cancer
CRC: colorectal carcinoma
HCC: hepatocelluar carcinoma
HBV: Hepatitis B virus
HCV: Hepatitis C virus
HBx: HBV X protein
ROS: reactive oxygen species
*H. pylori*: *Helicobacter pylori*
TLR: Toll-like receptor
GSK: glycogen synthase kinase
IECs: intestinal epithelial cells
IBD: inflammatory bowel disease
IL: interleukin
UC: ulcerative colitis
EECs: enteroendocrine cells
CgA: chromogranin A
5-ASA: 5-aminosalicylic acid
AP: acute pancreatitis
SAP: severe acute pancreatitis
TGF: transforming growth factor
BM-MSCs: Bone marrow mesenchymal stem cells
NAFLD: non-alcoholic fatty liver disease
NASH: non-alcoholic steatohepatitis
T2DM: type 2 diabetes mellitus
MBH: mediobasal hypothalamus
OA: Oleic acid
KCs: kupffer cells
HSCs: hepatic stellate cells
ECM: extracellular matrix
DNMT: DNA methyltransferase
HOTAIR: homeobox transcript antisense RNA
PSCs: pancreatic stellate cells
Mfn2: mitofusin-2.

## References

[B1] Alexandrov I. M. Ivshina M. Jung D. Y. Friedline R. Ko H. J. Xu M. (2012). Cytoplasmic polyadenylation element binding protein deficiency stimulates PTEN and Stat3 mRNA translation and induces hepatic insulin resistance. *PLoS. Genet* 8:e1002457. 10.1371/journal.pgen.1002457 22253608 PMC3257279

[B2] An J. Zheng L. Xie S. Yin F. Huo X. Guo J. (2016). Regulatory effects and mechanism of adenovirus-mediated PTEN gene on hepatic stellate cells. *Dig. Dis. Sci.* 61 1107–1120. 10.1007/s10620-015-3976-2 26660904

[B3] Bang Y. J. Kang Y. K. Ng M. Chung H. C. Wainberg Z. A. Gendreau S. (2019). A phase II, randomised study of mFOLFOX6 with or without the Akt inhibitor ipatasertib in patients with locally advanced or metastatic gastric or gastroesophageal junction cancer. *Eur. J. Cancer.* 108 17–24. 10.1016/j.ejca.2018.11.017 30592991

[B4] Bettstetter M. Berezowska S. Keller G. Walch A. Feuchtinger A. Slotta-Huspenina J. (2013). Epidermal growth factor receptor, phosphatidylinositol-3-kinase catalytic subunit/PTEN, and KRAS/NRAS/BRAF in primary resected esophageal adenocarcinomas: loss of PTEN is associated with worse clinical outcome. *Hum.Pathol.* 44 829–836. 10.1016/j.humpath.2012.08.005 23158210

[B5] Bian E. B. Huang C. Ma T. T. Tao H. Zhang H. Cheng C. (2012). DNMT1-mediated PTEN hypermethylation confers hepatic stellate cell activation and liver fibrogenesis in rats. *Toxicol. Appl.Pharmacol.* 264 13–22. 10.1016/j.taap.2012.06.022 22841775

[B6] Bononi A. Bonora M. Marchi S. Missiroli S. Poletti F. Giorgi C. (2013). Identification of PTEN at the ER and MAMs and its regulation of Ca(2+) signaling and apoptosis in a protein phosphatase-dependent manner. *Cell Death. Differ.* 20 1631–1643. 10.1038/cdd.2013.77 23811847 PMC3824603

[B7] Byrne C. D. (2009). Hypoxia and non-alcoholic fatty liver disease. *Clin. Sci. (Lond.)* 118 397–400. 10.1042/CS20090565 19900166

[B8] Cai S. W. Han Y. Wang G. P. (2018). miR-148a-3p exhaustion inhibits necrosis by regulating PTEN in acute pancreatitis. *Int. J.Clin. Exp.Pathol.* 11 5647–5657. 31949651 PMC6963085

[B9] Chen Y. Xiang W. Li X. Wang D. Qian C. (2019). Rosiglitazone prevents acute pancreatitis through inhibiting microRNA-26a expression. *Exp. Ther. Med.* 18 1246–1252. 10.3892/etm.2019.7711 31363368 PMC6614723

[B10] Cheng Y. Tian Y. Xia J. Wu X. Yang Y. Li X. (2017). The role of PTEN in regulation of hepatic macrophages activation and function in progression and reversal of liver fibrosis. *Toxicol. Appl. Pharmacol.* 317 51–62. 10.1016/j.taap.2017.01.005 28095306

[B11] Chia Y. C. Catimel B. Lio D. S. Ang C. S. Peng B. Wu H. (2015). The C-terminal tail inhibitory phosphorylation sites of PTEN regulate its intrinsic catalytic activity and the kinetics of its binding to phosphatidylinositol-4,5-bisphosphate. *Arch. Biochem. Biophys.* 587 48–60. 10.1016/j.abb.2015.10.004 26471078

[B12] Choi Y. J. Jung J. Chung H. K. Im E. Rhee S. H. (2013). PTEN regulates TLR5-induced intestinal inflammation by controlling Mal/TIRAP recruitment. *FASEB J.* 27 243–254. 10.1096/fj.12-217596 23038756 PMC3528317

[B13] Chung T. W. Lee Y. C. Kim C. H. (2004). Hepatitis B viral HBx induces matrix metalloproteinase-9 gene expression through activation of ERK and PI-3K/AKT pathways: involvement of invasive potential. *FASEB. J.* 18 1123–1125. 10.1096/fj.03-1429fje 15132991

[B14] Ciuffreda L. Di Sanza C. Cesta Incani U. Eramo A. Desideri M. Biagioni F. (2012). The mitogen-activatedproteinkinase (MAPK) cascadecontrolsphosphatase and tensinhomolog (PTEN) expressionthrough multiple mechanisms. *J. Mol. Med. (Berl.)* 90 667–679. 10.1007/s00109-011-0844-1 22215152

[B15] Clément S. Peyrou M. Sanchez-Pareja A. Bourgoin L. Ramadori P. Suter D. (2011). Down-regulation of phosphatase and tensin homolog by hepatitis C virus core 3a in hepatocytes triggers the formation of large lipid droplets. *Hepatology* 54 38–49. 10.1002/hep.24340 21465511

[B16] Clément S. Sobolewski C. Gomes D. Rojas A. Goossens N. Conzelmann S. (2019). Activation of the oncogenic miR-21-5p promotes HCV replication and steatosis induced by the viral core 3a protein. *Liver Int.* 39 1226–1236. 10.1111/liv.14112 30938910

[B17] de Brito O. M. Scorrano L. (2008). Mitofusin 2 tethers endoplasmic reticulum to mitochondria. *Nature* 456 605–610. 10.1038/nature07534 19052620

[B18] Dong Z. Li S. Si L. Ma R. Bao L. Bo A. (2020). Identification lncRNA LOC102551149/miR-23a-5p pathway in hepatic fibrosis. *Eur. J. Clin. Invest.* 50:e13243. 10.1111/eci.13243 32306379

[B19] Dong Z. Li S. Wang X. Si L. Ma R. Bao L. (2019). lncRNA GAS5 restrains CCl4-induced hepatic fibrosis by targeting miR-23a through the PTEN/PI3K/Akt signaling pathway. *Am. J. Physiol.Gastrointest. Liver Physiol.* 316 G539–G550. 10.1152/ajpgi.00249.2018 30735452

[B20] Du Y. Zhang Y. W. Pu R. Han X. Hu J. P. Zhang H. W. (2015). Phosphatase and tensin homologue genetic polymorphisms and their interactions with viral mutations on the risk of hepatocellular carcinoma. *Chin. Med. J. (Engl.)* 128 1005–1013. 10.4103/0366-6999.155057 25881591 PMC4832937

[B21] Dunbar C. E. High K. A. Joung J. K. Kohn D. B. Ozawa K. Sadelain M. (2018). Gene therapy comes of age. *Science* 359:6372. 10.1126/science.aan4672 29326244

[B22] Eng C. (2003). Constipation, polyps, or cancer? Let PTEN predict your future. *Am. J. Med. Genet. A* 122 315–322. 10.1002/ajmg.a.20477 14518069

[B23] Fang W. Guo J. Cao Y. Wang S. Pang C. Li M. (2016). MicroRNA-20a-5p contributes to hepatic glycogen synthesis through targeting p63 to regulate p53 and PTEN expression. *J. Cell Mol. Med.* 20 1467–1480. 10.1111/jcmm.12835 27019188 PMC4956936

[B24] Fragoso R. Barata J. T. (2015). Kinases, tails and more: regulation of PTEN function by phosphorylation. *Methods* 77 75–81. 10.1016/j.ymeth.2014.10.015 25448482

[B25] Gao Z. Q. Wang J. F. Chen D. H. Ma X. S. Wu Y. Tang Z. (2017). Long non-coding RNA GAS5 suppresses pancreatic cancer metastasis through modulating miR-32-5p/PTEN axis. *CellBiosci.* 7:66. 10.1186/s13578-017-0192-0 29225772 PMC5715988

[B26] Geng J. Huang X. Li Y. Xu X. Li S. Jiang D. (2016). Phosphatase and tensin homolog deleted on chromosome 10 contributes to phenotype transformation of fibroblasts in idiopathic pulmonary fibrosis via multiple pathways. *Exp. Biol. Med (Maywood)* 241 157–165. 10.1177/1535370215600100 26264443 PMC4935394

[B27] Geng W. Zhou G. Zhao B. Xiao Q. Li C. Fan S. (2020). Liquiritigenin suppresses the activation of hepatic stellate cells via targeting miR-181b/PTEN axis. *Phytomedicine* 66:153108. 10.1016/j.phymed.2019.153108 31790896

[B28] Geybels M. S. Fang M. Wright J. L. Qu X. Bibikova M. Klotzle B. (2017). PTEN loss is associated with prostate cancer recurrence and alterations in tumor DNA methylation profiles. *Oncotarget* 8 84338–84348. 10.18632/oncotarget.20940 29137428 PMC5663600

[B29] Gu J. Tamura M. Pankov R. Danen E. H. Takino T. Matsumoto K. (1999). Shc and FAK differentially regulate cell motility and directionality modulated by PTEN. *J. Cell Biol.* 146 389–403. 10.1083/jcb.146.2.389 10427092 PMC2156182

[B30] Gu T. Zhang Z. Wang J. Guo J. Shen W. H. Yin Y. (2011). CREB is a novel nuclear target of PTEN phosphatase. *Cancer Res.* 71 2821–2825. 10.1158/0008-5472.CAN-10-3399 21385900 PMC3105967

[B31] Guldberg P. thorStraten P. Birck A. Ahrenkiel V. Kirkin A. F. Zeuthen J. (1997). Disruption of the MMAC1/PTEN gene by deletion or mutation is a frequent event in malignant melanoma. *Cancer Res.* 57 3660–3663. 9288767

[B32] Guo J. Dou L. Meng X. Chen Z. Yang W. Fang W. (2017). Hepatic MiR-291b-3p mediated glucose metabolism by directly targeting p65 to upregulate PTEN expression. *Sci. Rep.* 7:39899. 10.1038/srep39899 28054586 PMC5214750

[B33] Ha H. L. Yu D. Y. (2010). HBx-induced reactive oxygen species activates hepatocellular carcinogenesis via dysregulation of PTEN/Akt pathway. *World J. Gastroenterol.* 16 4932–4937. 10.3748/wjg.v16.i39.4932 20954279 PMC2957601

[B34] Hao X. J. Xu C. Z. Wang J. T. Li X. J. Wang M. M. Gu Y. H. (2018). miR-21 promotes proliferation and inhibits apoptosis of hepatic stellate cells through targeting PTEN/PI3K/AKT pathway. *J.Recept. Signal.Transduct. Res.* 38 455–461. 10.1080/10799893.2019.1585452 31038023

[B35] Heinrich F. Chakravarthy S. Nanda H. Papa A. Pandolfi P. P. Ross A. H. (2015). The PTEN tumor suppressor forms homodimers in solution. *Structure* 23 1952–1957. 10.1016/j.str.2015.07.012 26299948 PMC4598300

[B36] Hernández-Trejo J. A. Suárez-Pérez D. Gutiérrez-Martínez I. Z. Fernandez-Vargas O. E. Serrano C. Candelario-Martínez A. A. (2016). The pro-inflammatory cytokines IFNγ/TNFα increase chromogranin A-positive neuroendocrine cells in the colonic epithelium. *Biochem. J.* 473 3805–3818. 10.1042/BCJ20160390 27538402

[B37] Hettinger K. Vikhanskaya F. Poh M. K. Lee M. K. de Belle I. Zhang J. T. (2007). C-Jun promotes cellular survival by suppression of PTEN. *Cell Death Differ.* 14 218–229. 10.1038/sj.cdd.4401946 16676006

[B38] Hopkins B. D. Fine B. Steinbach N. Dendy M. Rapp Z. Shaw J. (2013). A secreted PTEN phosphatase that enters cells to alter signaling and survival. *Science* 341 399–402. 10.1126/science.1234907 23744781 PMC3935617

[B39] Horie Y. Suzuki A. Kataoka E. Sasaki T. Hamada K. Sasaki J. (2004). Hepatocyte-specific Pten deficiency results in steatohepatitis and hepatocellular carcinomas. *J. Clin. Invest.* 113 1774–1783. 10.1172/JCI20513 15199412 PMC420505

[B40] Hou Z. Quan J. (2017). Hepatitis B virus X protein increases microRNA-21 expression and accelerates the development of hepatoma via the phosphatase and tensin homolog/phosphoinositide 3-kinase/protein kinase B signaling pathway. *Mol. Med. Rep.* 15 3285–3291. 10.3892/mmr.2017.6363 28339072

[B41] Howe C. Mitchell J. Kim S. J. Im E. Rhee S. H. (2019). Pten gene deletion in intestinal epithelial cells enhances susceptibility to *Salmonella Typhimurium* infection in mice. *J. Microbiol.* 57 1012–1018. 10.1007/s12275-019-9320-3 31555991

[B42] Im E. Jung J. Pothoulakis C. Rhee S. H. (2014). Disruption of Pten speeds onset and increases severity of spontaneous colitis in Il10(-/-) mice. *Gastroenterology* 147 667–679. 10.1053/j.gastro.2014.05.034 24882466 PMC4143453

[B43] Jeong S. H. Kim H. B. Kim M. C. Lee J. M. Lee J. H. Kim J. H. (2018). Hippo-mediated suppression of IRS2/AKT signaling prevents hepatic steatosis and liver cancer. *J. Clin. Invest.* 128 1010–1025. 10.1172/JCI95802 29400692 PMC5824861

[B44] Ji X. X. Ji X. J. Li Q. Q. Lu X. X. Luo L. (2018). Rosiglitazone reduces apoptosis and inflammation in lipopolysaccharide-induced human umbilical vein endothelial cells. *Med Sci. Monit.* 2018 6200–6207. 10.12659/MSM.910036 30185768 PMC6140784

[B45] Jia B. Yu D. Yu G. Cheng Y. Wang Y. Yi X. (2018). Naringenin improve hepatitis C virus infection induced insulin resistance by increase PTEN expression via p53-dependent manner. *Biomed. Pharmacother.* 103 746–754. 10.1016/j.biopha.2018.04.110 29684853

[B46] Jiang W. Liu J. Dai Y. Zhou N. Ji C. Li X. (2015). MiR-146b attenuates high-fat diet-induced non-alcoholic steatohepatitis in mice. *J. Gastroenterol. Hepatol.* 30 933–943. 25559563 10.1111/jgh.12878

[B47] Khalid A. Hussain T. Manzoor S. Saalim M. Khaliq S. (2017). PTEN: a potential prognostic marker in virus-induced hepatocellular carcinoma. *Tumour. Biol.* 39:1010428317705754. 10.1177/1010428317705754 28621226

[B48] Kim J. Kang H. S. Lee Y. J. Lee H. J. Yun J. Shin J. H. (2014). EGR1-dependent PTEN upregulation by 2-benzoyloxycinnamaldehyde attenuates cell invasion and EMT in colon cancer. *Cancer Lett.* 349 35–44. 10.1016/j.canlet.2014.03.025 24704156

[B49] Kleiner D. E. Makhlouf H. R. (2016). Histology of nonalcoholic fatty liver disease and nonalcoholic steatohepatitis in adults and children. *Clin. Liver Dis.* 20 293–312. 10.1016/j.cld.2015.10.011 27063270 PMC4829204

[B50] Kong G. Zhang J. Zhang S. Shan C. Ye L. Zhang X. (2011). Upregulated microRNA-29a by hepatitis B virus X protein enhances hepatoma cell migration by targeting PTEN in cell culture model. *PLoS One* 6:e19518. 10.1371/journal.pone.0019518 21573166 PMC3088678

[B51] Kong L. Wu Q. Zhao L. Ye J. Li N. Yang H. (2019). Effect of microRNA-27a-5p on apoptosis and inflammatory response of pancreatic acinar cells in acute pancreatitis by targeting PTEN. *J. Cell Biochem.* 120 15844–15850. 10.1002/jcb.28855 31106896

[B52] Kumar P. Raeman R. Chopyk D. M. Smith T. Verma K. Liu Y. (2018). Adiponectin inhibits hepatic stellate cell activation by targeting the PTEN/AKT pathway. *Biochim. Biophys. Acta. Mol. Basis. Dis.* 1864 3537–3545. 10.1016/j.bbadis.2018.08.012 30293572 PMC6529190

[B53] Kurtz J. R. Goggins J. A. McLachlan J. B. (2017). *Salmonella* infection: interplay between the bacteria and host immune system. *Immunol. Lett.* 190 42–50. 10.1016/j.imlet.2017.07.006 28720334 PMC5918639

[B54] Leclercq I. A. Da Silva Morais A. Schroyen B. Van Hul N. Geerts A. (2007). Insulin resistance in hepatocytes and sinusoidal liver cells: mechanisms and consequences. *J. Hepatol.* 47 142–156. 10.1016/j.jhep.2007.04.002 17512085

[B55] Lee D. Do I. G. Choi K. Sung C. O. Jang K. T. Choi D. (2012). The expression of phospho-AKT1 and phospho-MTOR is associated with a favorable prognosis independent of PTEN expression in intrahepatic cholangiocarcinomas. *Mod. Pathol.* 25 131–139. 10.1038/modpathol.2011.133 21874010

[B56] Lee D. Y. Jeyapalan Z. Fang L. Yang J. Zhang Y. Yee A. Y. (2010). Expression of versican 3’-untranslated region modulates endogenous microRNA functions. *PLoS One* 5:e13599. 10.1371/journal.pone.0013599 21049042 PMC2963607

[B57] Lee J. O. Yang H. Georgescu M. M. Di Cristofano A. Maehama T. Shi Y. (1999). Crystal structure of the PTEN tumour suppressor: implications for its phosphoinositide phosphatase activity and membrane association. *Cell* 99 323–334. 10.1016/s0092-8674(00)81663-3 10555148

[B58] Lee S. Y. Hur G. Y. Jung K. H. Jung H. C. Lee S. Y. Kim J. H. (2006). PPAR-gamma agonist increase gefitinib’s antitumor activity through PTEN expression. *Lung Cancer* 51 297–301. 10.1016/j.lungcan.2005.10.010 16386327

[B59] Lee Y. R. Chen M. Pandolfi P. P. (2018). The functions and regulation of the PTEN tumour suppressor: new modes and prospects. *Nat. Rev. Mol. Cell Biol.* 19 547–562. 10.1038/s41580-018-0015-0 29858604

[B60] Li H. Y. He H. C. Song J. F. Du Y. F. Guan M. Wu C. Y. (2020). Bone marrow-derived mesenchymal stem cells repair severe acute pancreatitis by secreting miR-181a-5p to target PTEN/Akt/TGF-β1 signaling. *Cell Signal.* 66:109436. 10.1016/j.cellsig.2019.109436 31654716

[B61] Li J. Yen C. Liaw D. Podsypanina K. Bose S. Wang S. I. (1997). PTEN, a putative protein tyrosine phosphatase gene mutated in human brain, breast, and prostate cancer. *Science* 275 1943–1947. 10.1126/science.275.5308.1943 9072974

[B62] Li P. Wang J. Zhao X. Ru J. Tian T. An Y. (2020). PTEN inhibition attenuates endothelial cell apoptosis in coronary heart disease via modulating the AMPK-CREB-Mfn2-mitophagy signaling pathway. *J. Cell Physiol.* 235 4878–4889. 10.1016/j.lfs.2020.117301 31654396

[B63] Li Z. Liu G. X. Liu Y. L. Chen X. Huang X. L. Gan H. T. (2013). Effect of adenovirus-mediated PTEN gene on ulcerative colitis-associated colorectal cancer. *Int. J. Colorectal Dis.* 2013 1107–1115. 10.1007/s00384-013-1678-9 23516074

[B64] Li Z. Zhang X. Jin T. Hao J. (2020). Nicotine promotes activation of human pancreatic stellate cells through inducing autophagy via α7nAChR-mediated JAK2/STAT3 signaling pathway. *Life Sci.* 243:117301. 10.1016/j.lfs.2020.117301 31953160

[B65] Liang H. Chen X. Yin Q. Ruan D. Zhao X. Zhang C. (2017). PTENβ is an alternatively translated isoform of PTEN that regulates rDNA transcription. *Nat. Commun.* 8:14771. 10.1038/ncomms14771 28332494 PMC5376652

[B66] Liang H. Wang X. Si C. Duan Y. Chen B. Liang H. (2020). Downregulation of miR-141 deactivates hepatic stellate cells by targeting the PTEN/AKT/mTOR pathway. *Int. J. Mol. Med.* 46 406–414. 10.3892/ijmm.2020.4578 32319536

[B67] Liaw D. Marsh D. J. Li J. Dahia P. L. Wang S. I. Zheng Z. (1997). Germline mutations of the PTEN gene in Cowden disease, an inherited breast and thyroid cancer syndrome. *Nat. Genet.* 16 64–67. 10.1038/ng0597-64 9140396

[B68] Lindsay Y. McCoull D. Davidson L. Leslie N. R. Fairservice A. Gray A. (2006). Localization of agonist-sensitive PtdIns(3,4,5)P3 reveals a nuclear pool that is insensitive to PTEN expression. *J. Cell Sci.* 119 5160–5168. 10.1242/jcs.000133 17158918

[B69] Ling C. Wang L. Wang Z. Xu L. Sun L. Yang H. (2015). A pathway-centric survey of somatic mutations in Chinese patients with colorectal carcinomas. *PLoS One* 10:e0116753. 10.1371/journal.pone.0116753 25617745 PMC4305320

[B70] Liu Y. Qi X. Zeng Z. Wang L. Wang J. Zhang T. (2017). CRISPR/Cas9-mediated p53 and Pten dual mutation accelerates hepatocarcinogenesis in adult hepatitis B virus transgenic mice. *Sci. Rep.* 7:2796. 10.1038/s41598-017-03070-8 28584302 PMC5459841

[B71] Lu J. Jeong H. W. Kong N. Yang Y. Carroll J. Luo H. R. (2009). Stem cell factor SALL4 represses the transcriptions of PTEN and SALL1 through an epigenetic repressor complex. *PLoS One* 4:e5577. 10.1371/journal.pone.0005577 19440552 PMC2679146

[B72] Maehama T. Dixon J. E. (1998). The tumor suppressor, PTEN/MMAC1, dephosphorylates the lipid second messenger, phosphatidylinositol 3,4,5-trisphosphate. *J. Biol. Chem.* 273 13375–13378. 10.1074/jbc.273.22.13375 9593664

[B73] Malik S. A. Acharya J. D. Mehendale N. K. Kamat S. S. Ghaskadbi S. S. (2019). Pterostilbene reverses palmitic acid mediated insulin resistance in HepG2 cells by reducing oxidative stress and triglyceride accumulation. *Free. Radic. Res.* 53 815–827. 10.1080/10715762.2019.1635252 31223033 PMC6675602

[B74] Managlia E. Katzman R. B. Brown J. B. Barrett T. A. (2013). Antioxidant properties of mesalamine in colitis inhibit phosphoinositide 3-kinase signaling in progenitor cells.Inflamm. *Bowel. Dis.* 19 2051–2060. 10.1097/MIB.0b013e318297d741 23867870 PMC8754500

[B75] Manning B. D. Cantley L. C. (2007). AKT/PKB signaling: navigating downstream. *Cell* 129 1261–1274. 10.1016/j.cell.2007.06.009 17604717 PMC2756685

[B76] Matsumoto M. Han S. Kitamura T. Accili D. (2006). Dual role of transcription factor FoxO1in controlling hepatic insulin sensitivity and lipid metabolism. *J. Clin. Invest.* 116 2464–2472. 10.1172/JCI27047 16906224 PMC1533874

[B77] Meng Z. Jia L. F. Gan Y. H. (2016). PTEN activation through K163 acetylation by inhibiting HDAC6 contributes to tumour inhibition. *Oncogene* 35 2333–2344. 10.1038/onc.2015.293 26279303

[B78] Milella M. Falcone I. Conciatori F. Cesta Incani U. Del Curatolo A. Inzerilli N. (2015). PTEN: multiple functions in human malignant tumors. *Front. Oncol.* 5:24. 10.3389/fonc.2015.00024 25763354 PMC4329810

[B79] Mitchell J. Kim S. J. Koukos G. Seelmann A. Veit B. Shepard B. (2018). Colonic inhibition of phosphatase and tensin homolog increases colitogenic bacteria, causing development of colitis in Il10-/- Mice. Inflamm. *Bowel. Dis.* 24 1718–1732. 10.1093/ibd/izy124 29788382 PMC6231371

[B80] Mohamed W. A. Salama R. M. Schaalan M. F. (2019). A pilot study on the effect of lactoferrin on Alzheimer’s disease pathological sequelae: impact of the p-Akt/PTEN pathway. *Biomed. Pharmacother.* 111 714–723. 10.1016/j.biopha.2018.12.118 30611996

[B81] Mueller S. Phillips J. Onar-Thomas A. Romero E. Zheng S. Wiencke J. K. (2012). PTEN promoter methylation and activation of the PI3K/Akt/mTOR pathway in pediatric gliomas and influence on clinical outcome. *Neuro. Oncol.* 14 1146–1152. 10.1093/neuonc/nos140 22753230 PMC3424210

[B82] Musso G. Gambino R. Cassader M. Pagano G. (2011). Meta-analysis: natural history of non-alcoholic fatty liver disease (NAFLD) and diagnostic accuracy of non-invasive tests for liver disease severity. *Ann. Med.* 43 617–649. 10.3109/07853890.2010.518623 21039302

[B83] Nalloor T. J. P. Kumar N. Narayanan K. Palanimuthu V. R. (2017). Long-term exposure to a butter-rich diet induces mild-to-moderate steatosis in Chang liver cells and Swiss albino mice models. *J. Basic Clin. Physiol.Pharmacol.* 28 257–265. 10.1515/jbcpp-2016-0058 28110314

[B84] Ni S. Wang H. Zhu X. Wan C. Xu J. Lu C. (2017). CBX7 suppresses cell proliferation, migration, and invasion through the inhibition of PTEN/Akt signaling in pancreatic cancer. *Oncotarget* 8 8010–8021. 10.18632/oncotarget.14037 28030829 PMC5352378

[B85] Niu X. Fu N. Du J. Wang R. Wang Y. Zhao S. (2016). miR-1273g-3p modulates activation and apoptosis of hepatic stellate cells by directly targeting PTEN in HCV-related liver fibrosis. *FEBS Lett.* 590 2709–2724. 10.1002/1873-3468.12309 27423040

[B86] Norén E. Almer S. Söderman J. (2017). Genetic variation and expression levels of tight junction genes identifies association between MAGI3 and inflammatory bowel disease. *BMC Gastroenterol.* 17:68. 10.1186/s12876-017-0620-y 28545409 PMC5445404

[B87] Norén E. Mellander M. R. Almer S. Söderman J. (2018). Genetic variation and gene expression levels of tight junction genes indicates relationships between PTEN as well as MAGI1 and microscopic colitis. *Dig. Dis. Sci.* 63 105–112. 10.1007/s10620-017-4857-7 29204743 PMC5760589

[B88] Papa A. Wan L. Bonora M. Salmena L. Song M. S. Hobbs R. M. (2014). Cancer-associated PTEN mutants act in a dominant negative manner to suppress PTEN protein function. *Cell* 157 595–610. 10.1016/j.cell.2014.03.027 24766807 PMC4098792

[B89] Park S. Ha Y. N. Dezhbord M. Lee A. R. Park E. S. Park Y. K. (2020). Suppression of hepatocyte nuclear factor 4 α by long-term infection of hepatitis B virus contributes to tumor cell proliferation. *Int. J. Mol. Sci.* 21:E948. 10.3390/ijms21030948 32023898 PMC7037729

[B90] Pitarresi J. R. Liu X. Avendano A. Thies K. A. Sizemore G. M. Hammer A. M. (2018). Disruption of stromal hedgehog signaling initiates RNF5-mediated proteasomal degradation of PTEN and accelerates pancreatic tumor growth. *Life Sci. Alliance* 1:e201800190. 10.26508/lsa.201800190 30456390 PMC6238420

[B91] Poliseno L. Salmena L. Zhang J. Carver B. Haveman W. J. Pandolfi P. P. (2010). A coding-independent function of gene and pseudogene mRNAs regulates tumour biology. *Nature* 465 1033–1038. 10.1038/nature09144 20577206 PMC3206313

[B92] Polytarchou C. Hommes D. W. Palumbo T. Hatziapostolou M. Koutsioumpa M. Koukos G. (2015). MicroRNA214 is Associated with progression of ulcerative colitis, and inhibition reduces development of colitis and colitis-associated cancer in mice. *Gastroenterology* 149 981–992. 10.1053/j.gastro.2015.05.057 26055138 PMC4584179

[B93] Qian Y. Y. Liu Z. S. Yan H. J. Yuan Y. F. Levenson A. S. Li K. (2018). Pterostilbene inhibits MTA1/HDAC1 complex leading to PTEN acetylation in hepatocellular carcinoma. *Biomed. Pharmacother.* 101 852–859. 10.1016/j.biopha.2018.03.022 29635894

[B94] Qiao Q. Li H. (2016). LncRNA FER1L4 suppresses cancer cell proliferation and cycle by regulating PTEN expression in endometrial carcinoma. *Biochem. Biophys. Res. Commun.* 478 507–512. 10.1016/j.bbrc.2016.06.160 27381864

[B95] Raftopoulou M. Etienne-Manneville S. Self A. Nicholls S. Hall A. (2004). Regulation of cell migration by the C2 domain of the tumor suppressor PTEN. *Science* 303 1179–1181. 10.1126/science.1092089 14976311

[B96] Ranjbar R. Hesari A. Ghasemi F. Sahebkar A. (2018). Expression of microRNAs and IRAK1 pathway genes are altered in gastric cancer patients with *Helicobacter pylori* infection. *J. Cell Biochem.* 119 7570–7576. 10.1002/jcb.27067 29797599

[B97] Roa I. de Toro G. Fernandez F. Game A. Munoz S. de Aretxabala X. (2015). Inactivation of tumor suppressor gene pten in early and advanced gallbladder cancer. *Diagn. Pathol.* 10:148. 10.1186/s13000-015-0381-2 26294099 PMC4546176

[B98] Roppenser B. Kwon H. Canadien V. Xu R. Devreotes P. N. Grinstein S. (2013). Multiple host kinases contribute to Akt activation during *Salmonella* infection. *PLoS One* 8:e71015. 10.1371/journal.pone.0071015 23990921 PMC3750030

[B99] Sanchez-Pareja A. Clément S. Peyrou M. Spahr L. Negro F. Rubbia-Brandt L. (2016). Phosphatase and tensin homolog is a differential diagnostic marker between nonalcoholic and alcoholic fatty liver disease. *World. J. Gastroenterol.* 22 3735–3745. 10.3748/wjg.v22.i14.3735 27076758 PMC4814736

[B100] Schneider E. Keppler R. Prawitt D. Steinwender C. Roos F. C. Thüroff J. W. (2011). Migration of renal tumor cells depends on dephosphorylation of Shc by PTEN. *Int. J. Oncol.* 38 823–831. 10.3892/ijo.2010.893 21206972

[B101] Seol J. E. Park I. H. Lee W. Kim H. Seo J. K. Oh S. H. (2015). Cowden syndrome with a novel germline PTEN mutation and an unusual clinical course. *Ann. Dermatol.* 27 306–309. 10.5021/ad.2015.27.3.306 26082588 PMC4466284

[B102] Shen S. M. Zhang C. Ge M. K. Dong S. S. Xia L. He P. (2019). PTENα and PTENβ promote carcinogenesis through WDR5 and H3K4 trimethylation. *Nat. Cell Boil.* 21 1436–1448. 10.1038/s41556-019-0409-z 31685992

[B103] Shen W. H. Balajee A. S. Wang J. Wu H. Eng C. Pandolfi P. P. (2007). Essential role for nuclear PTEN in maintaining chromosomal integrity. *Cell* 128 157–170. 10.1016/j.cell.2006.11.042 17218262

[B104] Shi Y. Wang J. Chandarlapaty S. Cross J. Thompson C. Rosen N. (2014). PTEN is a protein tyrosine phosphatase for IRS1. *Nat. Struct. Mol. Biol.* 21 522–527. 10.1038/nsmb.2828 24814346 PMC4167033

[B105] Song L. B. Li J. Liao W. T. Feng Y. Yu C. P. Hu L. J. (2009). The polycomb group protein Bmi-1 represses the tumor suppressor PTEN and induces epithelial-mesenchymal transition in human nasopharyngeal epithelial cells. *J. Clin. Invest.* 119 3626–3636. 10.1172/JCI39374 19884659 PMC2786794

[B106] Song M. S. Carracedo A. Salmena L. Song S. J. Egia A. Malumbres M. (2011). Nuclear PTEN regulates the APC-CDH1 tumor-suppressive complex in a phosphatase-independent manner. *Cell* 144 187–199. 10.1016/j.cell.2010.12.020 21241890 PMC3249980

[B107] Song Y. Li P. Qin L. Xu Z. Jiang B. Ma C. (2021). CUL4B negatively regulates Toll-like receptor-triggered proinflammatory responses by repressing Pten transcription. *Cell Mol. Immunol.* 2021 339–349. 10.1038/s41423-019-0323-0 31729464 PMC8026642

[B108] Stambolic V. MacPherson D. Sas D. Lin Y. Snow B. Jang Y. (2001). Regulation of PTEN transcription by p53. *Mol. Cell* 8 317–325. 10.1016/s1097-2765(01)00323-9 11545734

[B109] Sumita T. Ono H. Suzuki T. Sakai G. Inukai K. Katagiri H. (2014). Mediobasal hypothalamic PTEN modulates hepatic insulin resistance independently of food intake in rats. *Am. J. Physiol. Endocrinol. Metab.* 307 E47–E60. 10.1152/ajpendo.00361.2013 24824654

[B110] Sun Y. Yu M. Qu M. Ma Y. Zheng D. Yue Y. (2020). Hepatitis B virus-triggered PTEN/β-catenin/c-Mycsignalingenhances PD-L1 expression to promote immune evasion. *Am. J. Physiol. Gastrointest. Liver Physiol.* 318 G162–G173. 10.1152/ajpgi.00197.2019 31604033

[B111] Tu W. Yang Y. Song Y. Zhu W. (2019). Hepatitis B virus x protein accelerated the proliferation of hepatocellular carcinoma cell through lncRNA SNHG20/PTEN pathway. *J. Biochem.* 165 423–431. 10.1093/jb/mvy120 30690477

[B112] Um T. H. Kim H. Oh B. K. Kim M. S. Kim K. S. Jung G. (2011). Aberrant CpG island hypermethylation in dysplastic nodules and early HCC of hepatitis B virus-related human multistep hepatocarcinogenesis. *J. Hepatol.* 54 939–947. 10.1016/j.jhep.2010.08.021 21145824

[B113] Vazquez F. Grossman S. R. Takahashi Y. Rokas M. V. Nakamura N. Sellers W. R. (2001). Phosphorylation of the PTEN tail acts as an inhibitory switch by preventing its recruitment into a protein complex. *J. Biol. Chem.* 276 48627–48630. 10.1074/jbc.C100556200 11707428

[B114] Vazquez F. Ramaswamy S. Nakamura N. Sellers W. R. (2000). Phosphorylation of the PTEN tail regulates protein stability and function. *Mol. Cell Biol.* 20 5010–5018. 10.1128/mcb.20.14.5010-5018.2000 10866658 PMC85951

[B115] Vezza T. Rodríguez-Nogales A. Algieri F. Utrilla M. P. Rodriguez-Cabezas M. E. Galvez J. (2016). Flavonoids in inflammatory bowel disease: a review. *Nutrients* 8:211. 10.3390/nu8040211 27070642 PMC4848680

[B116] Vukelić I. Detel D. Pučar L. B. Potočnjak I. Buljević S. Domitrović R. (2018). Chlorogenic acid ameliorates experimental colitis in mice by suppressing signaling pathways involved in inflammatory response and apoptosis. *Food Chem. Toxicol.* 121 140–150. 10.1016/j.fct.2018.08.061 30165128

[B117] Wadhwa R. Song S. Lee J. S. Yao Y. Wei Q. Ajani J. A. (2013). Gastric cancer-molecular and clinical dimensions. *Nat. Rev. Clin. Oncol.* 10 643–655. 10.1038/nrclinonc.2013.170 24061039 PMC3927982

[B118] Walker S. M. Leslie N. R. Perera N. M. Batty I. H. Downes C. P. (2004). The tumour-suppressor function of PTEN requires an N-terminal lipid-binding motif. *Biochem. J.* 15 301–307. 10.1042/BJ20031839 14711368 PMC1224073

[B119] Wang J. Xu W. He Y. Xia Q. Liu S. (2018). LncRNA MEG3 impacts proliferation, invasion, and migration of ovarian cancer cells through regulating PTEN. *Inflamm. Res.* 67 927–936. 10.1007/s00011-018-1186-z 30310931

[B120] Wang L. Cho Y. L. Tang Y. Wang J. Park J. E. Wu Y. (2018). PTEN-L is a novel protein phosphatase for ubiquitin dephosphorylation to inhibit PINK1-Parkin-mediated mitophagy. *Cell Res.* 28 787–802. 10.1038/s41422-018-0056-0 29934616 PMC6082900

[B121] Wang S. Wang L. Dou L. Guo J. Fang W. Li M. (2016). MicroRNA 152 regulates hepatic glycogenesis by targeting PTEN. *FEBS. J.* 283 1935–1946. 10.1111/febs.13713 26996529

[B122] Wang Z. Li N. Wang B. Lin J. (2015a). Nonalcoholic fatty liver disease progression in rats is accelerated by splenic regulation of liver PTEN/AKT. *Saudi. J. Gastroenterol.* 21 232–238. 10.4103/1319-3767.161641 26228367 PMC4542422

[B123] Wang Z. Wu N. Tesfaye A. Feinstone S. Kumar A. (2015b). HCV infection-associated hepatocellular carcinoma in humanized mice. *Infect. Agent Cancer* 10:24. 10.1186/s13027-015-0018-9 26217396 PMC4515941

[B124] Williams A. J. Doherty E. S. Hart M. H. Grider D. J. (2018). Diffuse gastric ganglioneuromatosis: novel presentation of PTEN hamartoma syndrome-case report and review of gastric ganglioneuromatous proliferations and a novel PTEN gene mutation. *Case. Rep. Med.* 2018:4319818. 10.1155/2018/4319818 29770149 PMC5889855

[B125] Worby C. A. Dixon J. E. (2014). PTEN. *Annu. Rev.Biochem.* 83 641–669. 10.1146/annurev-biochem-082411-113907 24905788

[B126] Wu H. T. Chen W. Cheng K. C. Ku P. M. Yeh C. H. Cheng J. T. (2012). Oleic acid activates peroxisome proliferator-activated receptor δ to compensate insulin resistance in steatotic cells. *J. Nutr. Biochem.* 23 1264–1270. 10.1016/j.jnutbio.2011.07.006 22209682

[B127] Wu Q. Li Z. Liu Q. (2017a). Treatment with PTEN-Long protein inhibits hepatitis C virus replication. *Virology* 2017 1–8. 10.1016/j.virol.2017.08.002 28783500

[B128] Wu Q. Li Z. Mellor P. Zhou Y. Anderson D. H. Liu Q. (2017b). The role of PTEN–HCV core interaction in hepatitis C virus replication. *Sci. Rep.* 7:3695. 10.1038/s41598-017-03052-w 28623358 PMC5473856

[B129] Wu S. M. Li T. H. Yun H. Ai H. W. Zhang K. H. (2019). miR-140-3p knockdown suppresses cell proliferation and fibrogenesis in hepatic stellate cells via PTEN-mediated AKT/mTOR signaling. *Yonsei. Med. J.* 60 561–569. 10.3349/ymj.2019.60.6.561 31124340 PMC6536388

[B130] Wu W. Yang J. Feng X. Wang H. Ye S. Yang P. (2013). MicroRNA-32 (miR-32) regulates phosphatase and tensin homologue (PTEN) expression and promotes growth, migration, and invasion in colorectal carcinoma cells. *Mol. Cancer* 12 30. 10.1186/1476-4598-12-30 23617834 PMC3653742

[B131] Wu X. Chen K. Williams K. J. (2020). An oxide transport chain essential for balanced insulin action. *Atherosclerosis* 298 42–51. 10.1016/j.atherosclerosis.2020.02.006 32171979

[B132] Wu Y. Song Y. Xiong Y. Wang X. Xu K. Han B. (2017c). MicroRNA-21 (Mir-21) promotes cell growth and invasion by repressing tumor suppressor PTEN in colorectal cancer. *Cell Physiol. Biochem.* 43 945–958. 10.1159/000481648 28957811

[B133] Xie S. R. An J. Y. Zheng L. B. Huo X. X. Guo J. Shih D. (2017). Effects and mechanism of adenovirus-mediated phosphatase and tension homologue deleted on chromosome ten gene on collagen deposition in rat liver fibrosis. *World J. Gastroenterol.* 23 5904–5912. 10.3748/wjg.v23.i32.5904 28932082 PMC5583575

[B134] Xiong Y. Qiu J. Li C. Qiu Y. Guo L. Liu Y. (2018). Fortunellin-induced modulation of phosphatase and tensin homolog by MicroRNA-374a decreases inflammation and maintains intestinal barrier function in Colitis. *Front. Immunol.* 26:83. 10.3389/fimmu.2018.00083 29472916 PMC5810275

[B135] Xu M. Wang G. Zhou H. Cai J. Li P. Zhou M. (2017). TGF-β1-miR-200a-PTEN induces epithelial-mesenchymal transition and fibrosis of pancreatic stellate cells. *Mol. Cell Biochem.* 431 161–168. 10.1007/s11010-017-2988-y 28281184

[B136] Xu W. Huang Y. Yang Z. Hu Y. Shu X. Xie C. (2018). *Helicobacter pylori* promotes gastric epithelial cell survival through the PLK1/PI3K/Akt pathway. *Onco Targets Ther.* 11 5703–5713. 10.2147/OTT.S164749 30254463 PMC6140703

[B137] Xue R. Yang J. Wu J. Meng Q. Hao J. (2017). Coenzyme Q10 inhibits the activation of pancreatic stellate cells through PI3K/AKT/mTOR signaling pathway. *Oncotarget* 8 92300–92311. 10.18632/oncotarget.21247 29190916 PMC5696182

[B138] Yan B. Cheng L. Jiang Z. Chen K. Zhou C. Sun L. (2018). Resveratrol inhibits ROS-promoted activation and glycolysis of pancreatic stellate cells via suppression of miR-21. *Oxid. Med. Cell. Longev.* 2018:1346958. 10.1155/2018/1346958 29854071 PMC5944235

[B139] Yan Z. Zang B. Gong X. Ren J. Wang R. (2020). MiR-214-3p exacerbates kidney damages and inflammation induced by hyperlipidemic pancreatitis complicated with acute renal injury. *Life Sci.* 241:117118. 10.1016/j.lfs.2019.117118 31790686

[B140] Yang T. S. Yang X. H. Chen X. Wang X. D. Hua J. Zhou D. L. (2014). MicroRNA-106b in cancer-associated fibroblasts from gastric cancer promotes cell migration and invasion by targeting PTEN. *FEBS Lett.* 588 2162–2169. 10.1016/j.febslet.2014.04.050 24842611

[B141] Yang Y. L. Wang F. S. Li S. C. Tiao M. M. Huang Y. H. (2017). MicroRNA-29a alleviates bile duct ligation exacerbation of hepatic fibrosis in mice through epigenetic control of methyltransferases. *Int. J. Mol. Sci.* 18:192. 10.3390/ijms18010192 PMC5297823 28106784

[B142] Yang Z. Cao X. Xu W. Xie C. Chen J. Zhu Y. (2018). Phosphorylation of phosphatase and tensin homolog induced by *Helicobacter pylori* promotes cell invasion by activation of focal adhesion kinase. *Oncol. Lett.* 15 1051–1057. 10.3892/ol.2017.7430 29399165 PMC5772772

[B143] Yang Z. Xie C. Xu W. Liu G. Cao X. Li W. (2015). Phosphorylation and inactivation of PTEN at residues Ser380/Thr382/383 induced by *Helicobacter* pyloripromotesgastricepithelialcellsurvivalthrough PI3K/Aktpathway. *Oncotarget* 6 31916–31926. 10.18632/oncotarget.5577 26376616 PMC4741650

[B144] Ying H. Elpek K. G. Vinjamoori A. Zimmerman S. M. Chu G. C. Yan H. (2011). PTEN is a major tumor suppressor in pancreatic ductal adenocarcinoma and regulates an NF-κB-cytokine network. *Cancer Discov.* 1 158–169. 10.1158/2159-8290.CD-11-0031 21984975 PMC3186945

[B145] Yoshimi A. Goyama S. Watanabe-Okochi N. Yoshiki Y. Nannya Y. Nitta E. (2011). Evi1 represses PTEN expression and activates PI3K/AKT/mTOR via interactions with polycomb proteins. *Blood* 117 3617–3628. 10.1182/blood-2009-12-261602 21289308

[B146] Yu F. Chen B. Dong P. Zheng J. (2017). HOTAIR epigenetically modulates PTEN expression via MicroRNA-29b: a novel mechanism in regulation of liver fibrosis. *Mol. Ther.* 25 205–217. 10.1016/j.ymthe.2016.10.015 28129115 PMC5363197

[B147] Yu F. Lin Z. Zheng J. Gao S. Lu Z. Dong P. (2014). Suppression of collagen synthesis by Dicer gene silencing in hepatic stellate cells. *Mol. Med. Rep.* 9 707–714. 10.3892/mmr.2013.1866 24337369

[B148] Yu G. Chen X. Chen S. Ye W. Hou K. Liang M. (2016). MiR-19a, miR-122 and miR-223 are differentially regulated by hepatitis B virus X protein and involve in cell proliferation in hepatoma cells. *J. Transl. Med.* 14:122. 10.1186/s12967-016-0888-7 27150195 PMC4858919

[B149] Yu Q. Tan R. Z. Gan Q. Zhong X. Wang Y. Q. Zhou J. (2017). A novel rat model of nonalcoholic fatty liver disease constructed through CRISPR/Cas-based hydrodynamic injection. *Mol. Biotechnol.* 59 365–373. 10.1007/s12033-017-0025-8 28695481

[B150] Zhang J. Ning X. Cui W. Bi M. Zhang D. Zhang J. (2015). Transforming growth factor (TGF)-β-induced microRNA-216a promotes acute pancreatitis via Akt and TGF-β pathway in mice. *Dig. Dis. Sci.* 60 127–135. 10.1007/s10620-014-3261-9 25501921

[B151] Zhang L. Li X. Zhang N. Yang X. Hou T. Fu W. (2020). WDFY2 potentiates hepatic insulin sensitivity and controls endosomal localization of the insulin receptor and IRS1/2. *Diabetes* 69 1887–1902. 10.2337/db19-0699 32641353

[B152] Zhang L. L. Liu J. Lei S. Zhang J. Zhou W. Yu H. G. (2014). PTEN inhibits the invasion and metastasis of gastric cancer via downregulation of FAK expression. *Cell. Signal.* 26 1011–1020. 10.1016/j.cellsig.2014.01.025 24486402

[B153] Zhang Q. Liang H. Zhao X. Zheng L. Li Y. Gong J. (2021). PTENε suppresses tumor metastasis through regulation of filopodia formation. *EMBO J.* 10.15252/embj.2020105806 [Epub ahead of print] 33755220 PMC8126949

[B154] Zhang W. Fu X. Xie J. Pan H. Han W. Huang W. (2021). miR-26a attenuates colitis and colitis-associated cancer by targeting the multiple intestinal inflammatory pathways. *Mol. Ther. Nucleic Acids* 24 264–273. 10.1016/j.omtn.2021.02.029 33815939 PMC7985669

[B155] Zhang X. Jia X. Mei L. Zheng M. Yu C. Ye M. (2016). Global DNA methylation and PTEN hypermethylation alterations in lung tissues from human silicosis. *J.Thorac. Dis.* 8 2185–2195. 10.21037/jtd.2016.07.21 27621875 PMC4999681

[B156] Zhang X. Jin T. Huang X. Liu X. Liu Z. Jia Y. (2018). Effects of the tumor suppressor PTEN on biological behaviors of activated pancreatic stellate cells in pancreatic fibrosis. *Exp. Cell Res.* 373 132–144. 10.1016/j.yexcr.2018.10.005 30321515

[B157] Zhao H. He Z. Huang D. Gao J. Gong Y. Wu H. (2016). Infusion of bone marrow mesenchymal stem cells attenuates experimental severe acute pancreatitis in rats. *Stem Cells Int.* 2016:7174319. 10.1155/2016/7174319 27721836 PMC5046031

[B158] Zheng J. Wu C. Lin Z. Guo Y. Shi L. Dong P. (2014). Curcumin up-regulates phosphatase and tensin homologue deleted on chromosome 10 through microRNA-mediated control of DNA methylation–a novel mechanism suppressing liver fibrosis. *FEBS. J.* 281 88–103. 10.1111/febs.12574 24138392

[B159] Zhu Y. Hoell P. Ahlemeyer B. Krieglstein J. (2006). PTEN: a crucial mediatorofmitochondria-dependentapoptosis. *Apoptosis* 11 197–207. 10.1007/s10495-006-3714-5 16502258

[B160] Zuo M. L. Wang A. P. Tian Y. Mao L. Song G. L. Yang Z. B. (2019). Oxymatrine ameliorates insulin resistance in rats with type 2 diabetes by regulating the expression of KSRP, PETN, and AKT in the liver. *J. Cell Biochem.* 120 16185–16194. 10.1002/jcb.28898 31087709

